# Zinc chloride is effective as an antibiotic in biofilm prevention following septoplasty

**DOI:** 10.1038/s41598-023-35069-9

**Published:** 2023-05-23

**Authors:** Noa Noach, Eran Lavy, Ram Reifen, Michael Friedman, David Kirmayer, Einat Zelinger, Amit Ritter, Dan Yaniv, Ella Reifen

**Affiliations:** 1 The Institute of Biochemistry, Food Science and Nutrition. The Robert H. Smith Faculty of Agriculture, Food & Environment, The Hebrew University of Jerusalem, Rehovot, Israel; 2 The Koret School of Veterinary Medicine. The Robert H. Smith Faculty of Agriculture, Food & Environment, The Hebrew University of Jerusalem, Rehovot, Israel; 3 FACSI-Faculty of Agriculture Center for Scientific Imaging. The Robert H. Smith Faculty of Agriculture, Food & Environment, The Hebrew University of Jerusalem, Rehovot, Israel; 4 The School of Pharmacy, The Faculty of Medicine, Ein Kerem Campus, The Hebrew University of Jerusalem, Jerusalem, Israel; 5 Department of Otolaryngology, Head and Neck Surgery, Rabin Medical Center, Petah Tikva, Israel

**Keywords:** Microbiology, Antimicrobials, Biofilms, Pathogens

## Abstract

Biofilm-state bacterial infections associated with inserted medical devices constitute a massive health and financial problem worldwide. Although bacteria exhibit significantly lower susceptibility to antibiotics in the biofilm state, the most common treatment approach still relies on antibiotics, exacerbating the phenomenon of antibiotic-resistant bacteria. In this study, we aimed to assess whether ZnCl_2_ coating of intranasal silicone splints (ISSs) can reduce the biofilm infections associated with the insertion of these devices and prevent the overuse of antibiotics while minimizing waste, pollution and costs. We tested the ability of ZnCl_2_ to prevent biofilm formation on ISS both in vitro and in vivo by using the microtiter dish biofilm formation assay, crystal violet staining, and electron and confocal microscopy. We found a significant decrease in biofilm formation between the treatment group and the growth control when ZnCl_2_-coated splints were placed in patients’ nasal flora. According to these results, infections associated with ISS insertion may be prevented by using ZnCl_2_ coating, thereby obviating the overuse and abuse of antibiotics.

## Introduction

Medical device-associated infections are largely responsible for increased morbidity and mortality in patients and for severe financial losses to healthcare services^1^. Currently, a wide range of medical processes in almost all fields of medicine require the insertion of foreign bodies into the patient's body that might cause severe infections, referred to as foreign-body-related infections (FBRIs)^2^. The vast majority of these infections are caused by bacteria, particularly in the biofilm state, that colonize the surfaces of inserted medical foreign devices. Infections of medical devices typically occur during the implantation procedure as a result of the inoculation of small numbers of bacteria that originate from the patient's skin or mucous membrane. The inoculation may, however, also originate from the hands of the surgical staff, from contaminated disinfectants and from the hospital’s environment^2,3^.

Septal surgery (septoplasty), a surgical procedure to correct a deviated nasal septum, is one of the most common surgical procedures in facial surgery^4–7^. ISSs are often used in septoplasty for stabilizing the operated septum and remaining cartilage, promoting mucosal healing, and preventing nasal synechia^8–10^. The most common microorganisms associated with nasal splint-related infections are *Staphylococcus aureus*, *Pseudomonas aeruginosa*, *Klebsiella pneumonia* and *Enterobacter aerogenes*^11,12^, mainly in a biofilm form.

A biofilm is an organized aggregate of microorganisms embedded within a self-produced matrix of extracellular polymeric substances (EPS) that is attached to a biotic or abiotic surface and formed in a complex and dynamic process of reversible attachment, irreversible attachment, growth and differentiation, and dissemination^13^. The biofilm state streamlines the exchange of plasmids among bacteria, some of which often contain multidrug-resistant coding genes. Therefore, biofilms also pose a danger of increasing antibiotic-resistant bacteria^14,15^.

The basic principle of device-associated infection control relies on prevention rather than treatment, in view of the high feasibility of a rapid worsening of the infection following the development of biofilm^14,16^. Currently, antibiotics are the most common treatment for medical device-associated infections^2,17^. However, due to the bacteria’s reduced susceptibility to antibiotics in the biofilm state^18^, the treatment of these infections often resorts to the removal of the implanted device and, if possible or needed, its replacement with a new device^2,19^. Currently, the main course of action after nasal surgery is the administration of systemic prophylactic antibiotics, although it is not universally agreed upon as effective^11,20^. Over 70% of bacterial infections are known to be resistant to one or more of the antibiotics generally used for infection eradication^21^. This has resulted in a massive search for alternative antimicrobial substances and alternative treatment approaches to prevent bacterial and biofilm-state bacterial infections^2,16,22^. An important strategy for preventing the formation of biofilms related to medical devices is surface modification—applying antibacterial or antiadhesion agents onto the medical device’s surface^22,23^. Given the abovementioned disadvantages of antibiotics, the current trend is the development of non-antibiotic coatings for medical devices, such as metal-based coatings^24,25^.

Metals have been used as antibacterial agents for decades in daily life, industry, agriculture, and healthcare. Certain metals are crucial for the biochemistry of life in all organisms, fulfilling cellular functions that cannot be replaced by organic molecules, and are therefore recognized as essential metals, of which the most common antibacterial elements are zinc, silver and copper^26–29^. When presented in high concentrations, however, zinc is also toxic to bacterial cells due to the blockage of essential reactions in them^30,31^. Zinc is known to have a wide range of antibacterial and antibiofilm activities, affecting many bacterial strains by interfering with various cellular processes and effectively inhibiting their growth^30,31^. Zinc chloride (ZnCl_2_) has already been shown to have antibacterial and antibiofilm activity^32,33^, although to the best of our knowledge, it has not been utilized for the prevention or treatment of medical device-related infections.

We hypothesized that the use of ZnCl_2_ coating for medical devices will reduce the bacterial infections associated with the insertion of these devices and prevent the unnecessary use of antibiotics while minimizing waste, pollution and costs. This study focused on nasal splints as a model medical device application of the ZnCl_2_ coating, and our aims were therefore the following: (1) to investigate the antibacterial and antibiofilm activity of ZnCl_2_ against clinically isolated bacteria in vitro; (2) to investigate the antibacterial and antibiofilm activity of ZnCl_2_-coated splints in vitro; (3) to examine the effect of prolonged presence of ZnCl_2_, ruling out possible local or systemic toxicity in vivo*;* and (4) to assess the efficacy of ZnCl_2_-coated splints in vivo (preliminary studies).

## Results

### Susceptibility of planktonic and biofilm cells to ZnCl_2_

*Staphylococcus aureus* strains were found to be susceptible to ZnCl_2_ for both planktonic and biofilm cells and showed a significant decrease in viability when presented with ZnCl_2_ concentrations ranging from 0.9 to 7.0 mM (Table 1). For planktonic cells, the minimum inhibitory concentration (MIC) value was obtained at a ZnCl_2_ concentration of 1.2 mM for both strains, and minimum bactericidal concentration (MBC) values ranged from 5.0 to 7.0 mM. For biofilm cells, biofilm preventive concentration (BPC) values ranged from 0.9 to 1.0 mM. *E. aerogenes* strains were found to be susceptible to ZnCl_2_ for both biofilm and planktonic cells. BPC was obtained at 2.0 mM for both strains, MIC values ranged from 3.0 to 4.0 mM, and MBC was not found within the tested range. For *P. aeruginosa* strains, BPC was obtained at 4.0 mM for both strains. No MIC or MBC values were found, however, for planktonic cells within the tested range.

**Table 1:** Inhibitory, bactericidal and biofilm prevention concentrations of zinc chloride for the examined microorganisms.

Microorganism	Isolate ID	Zinc-chloride solution concentration (mM)		
		MIC	MBC	BPC
*Staphylococcus aureus*	43021480	1.2	5.0	0.9
*Staphylococcus aureus*	43021481	1.2	7.0	1.0
*Enterobacter aerogenes*	43013795	3.0	NF	2.0
*Enterobacter aerogenes*	43013796	4.0	NF	2.0
*Pseudomonas aeruginosa*	43013791	NF	NF	4.0
*Pseudomonas aeruginosa*	43013792	NF	NF	4.0

*MIC* minimum inhibitory concentration, *MBC* minimum bactericidal concentration, *BPC* biofilm preventive concentration, *NF* not found within the tested range of ZnCl_2_ concentrations.

### Biofilm mass inhibition of clinical bacteria on the coated splints in vitro

Biofilm formation on the ZnCl_2_-coated splints of three different clinical bacterial strains, *P. aeruginosa*, *E. aerogenes* and *S. aureus,* was significantly (p < 0.001) inhibited by 55–70% in comparison with the positive growth control of non-ZnCl_2_-coated splints (data not shown). Inhibition was successfully achieved and was consistent across 168 h of incubation, with no significant differences.

### Confocal laser scanning microscopy (CLSM) quantification of biofilm mass inhibition of the clinical bacteria on the coated splints in vitro

**Figure 1:**
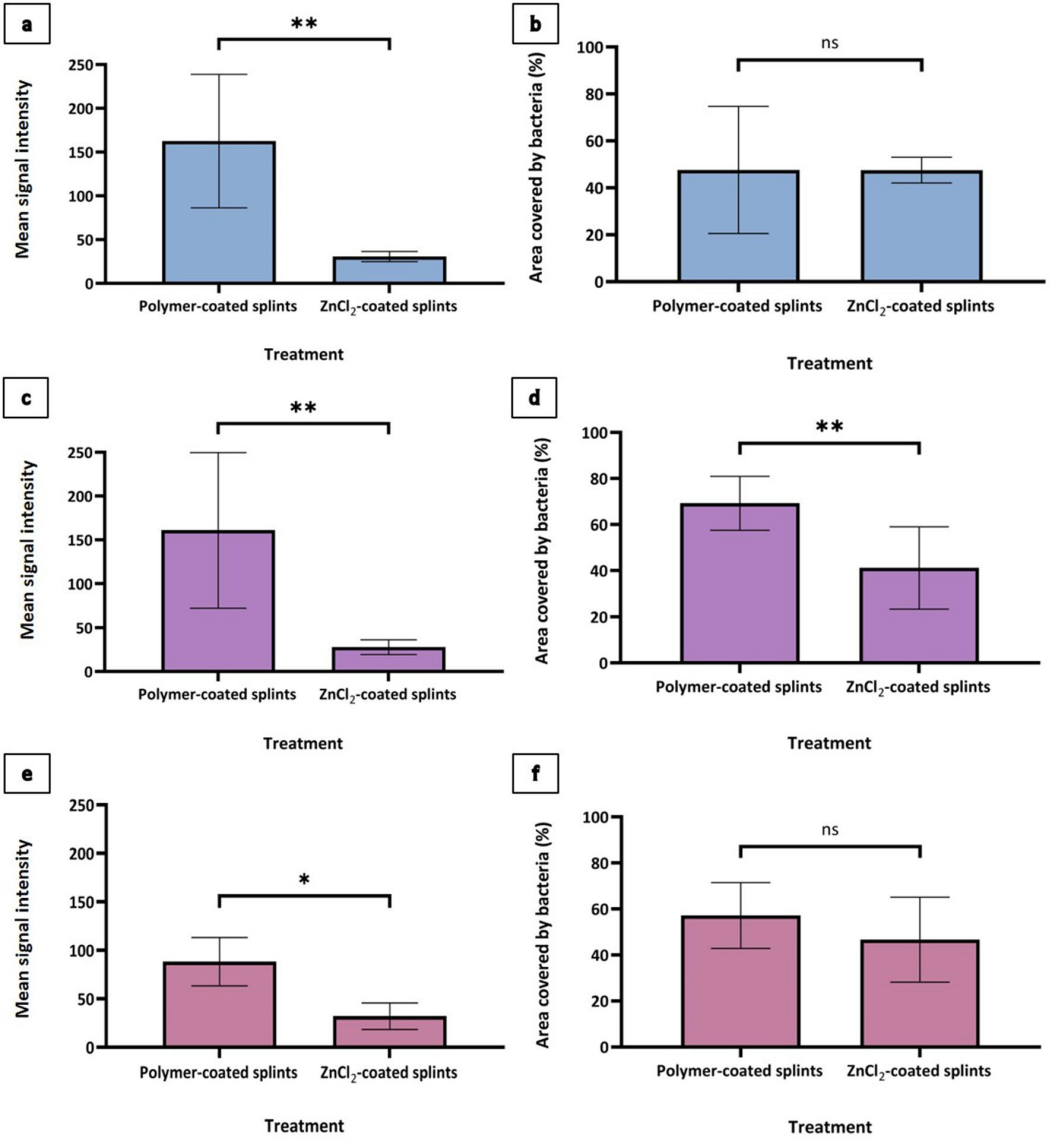
Area covered by bacterial biofilm (%) and biofilm mean signal intensity in vitro, as evaluated by CLSM. *Staphylococcus aureus* (**a**,**b**), *Pseudomonas aeruginosa* (**c**,**d**) and *Enterobacter aerogenes* (**e**,**f**) biofilms were grown on ZnCl_2_-coated splints and on non-ZnCl_2_-coated splints without ZnCl_2_ as positive growth controls. Pieces of these splints were stained with propidium iodide (PI) and evaluated by CLSM for the area of each piece covered by bacterial biofilm (right column) and for the mean signal intensity, quantifying the amount of biofilm formed on the splint surface (left column). P-values are provided by unpaired t-test (*p 0.05; **p < 0.01; ***p < 0.001).

**Figure 2:**
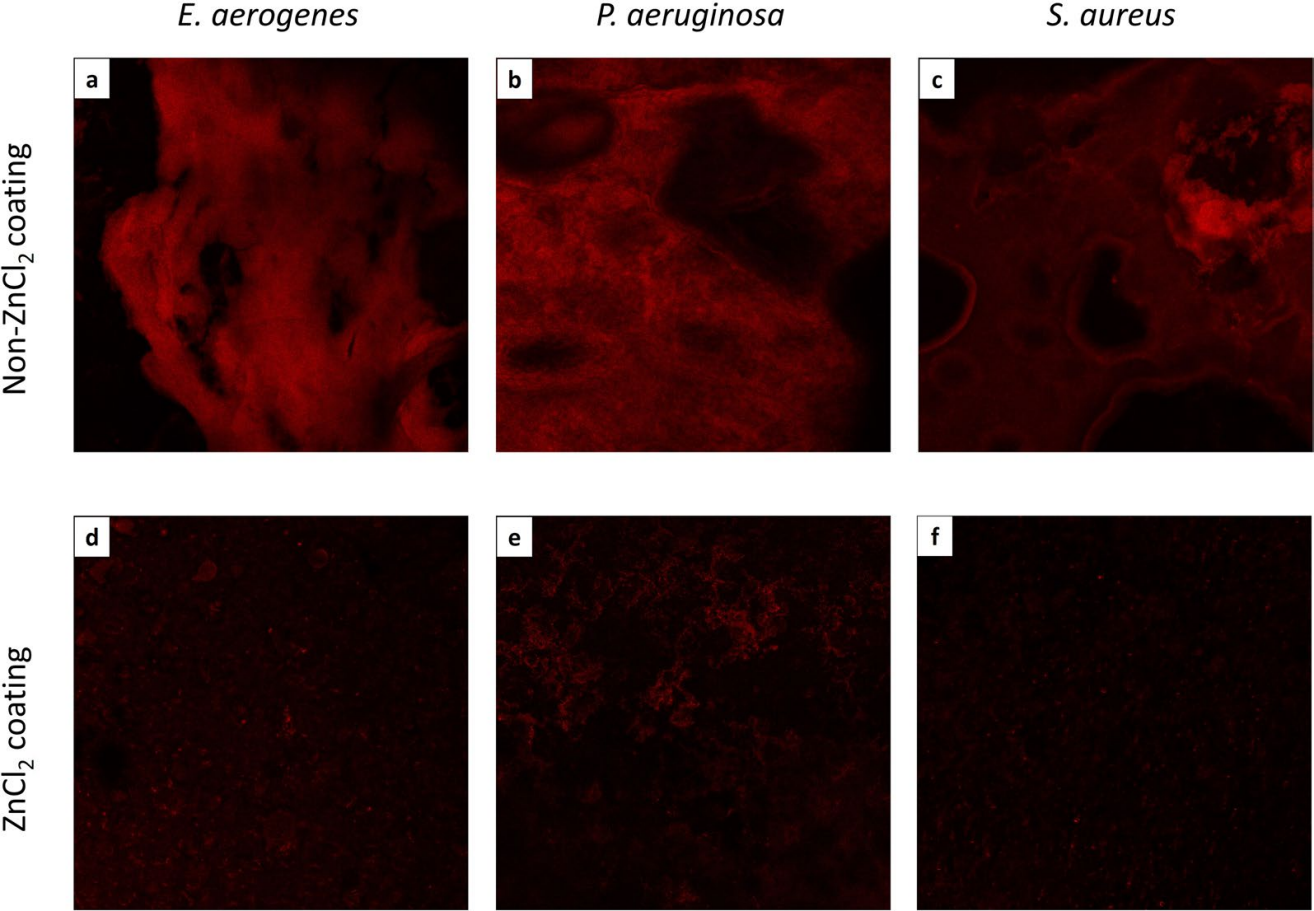
Confocal production of bacterial biofilm in vitro, as evaluated by CLSM*. Enterobacter aerogenes* (**a**,**d**), *Pseudomonas aeruginosa* (**b**,**e**) and *Staphylococcus aureus* (**c**,**f**) biofilms were grown on ZnCl_2_-coated splints and on non-ZnCl_2_-coated splints as positive growth controls. Pieces of these splints were stained with propidium iodide (PI) and evaluated by CLSM.

CLSM was used to evaluate biofilm formation of three different clinical bacterial strains, *S. aureus, P. aeruginosa* and *E. aerogenes*, onto ZnCl_2_-coated splints and onto non-ZnCl_2_-coated splints as a positive growth control. Biofilm growth on the splint surfaces was evaluated using two methods: first, by the area covered by bacteria (%), quantifying the bacteria's ability to attach to the splint surface, and second, by the mean signal intensity, quantifying the amount of biofilm mass. From the measured area covered by bacteria (%) (Fig. 1, right column), in both *S. aureus* and *E. aerogenes*, no significant differences were found in the ability to attach to the splint surfaces between ZnCl_2_-coated splints and non-ZnCl_2_-coated splints. A significant decrease (p < 0.01) in this ability was exhibited in *P. aeruginosa*. All strains exhibited a significant decrease (*S. aureus* and *P. aeruginosa*: p < 0.01, *E. aerogenes*: p < 0.05) in the mean biofilm mass on the ZnCl_2_-coated splints in comparison with the polymer-only-coated splints (Fig. 1, left column and Fig. 2).

### Biofilm mass inhibition in postinsertion ZnCl_2_-coated splints, evaluated by CLSM, in vivo

**Figure 3:**
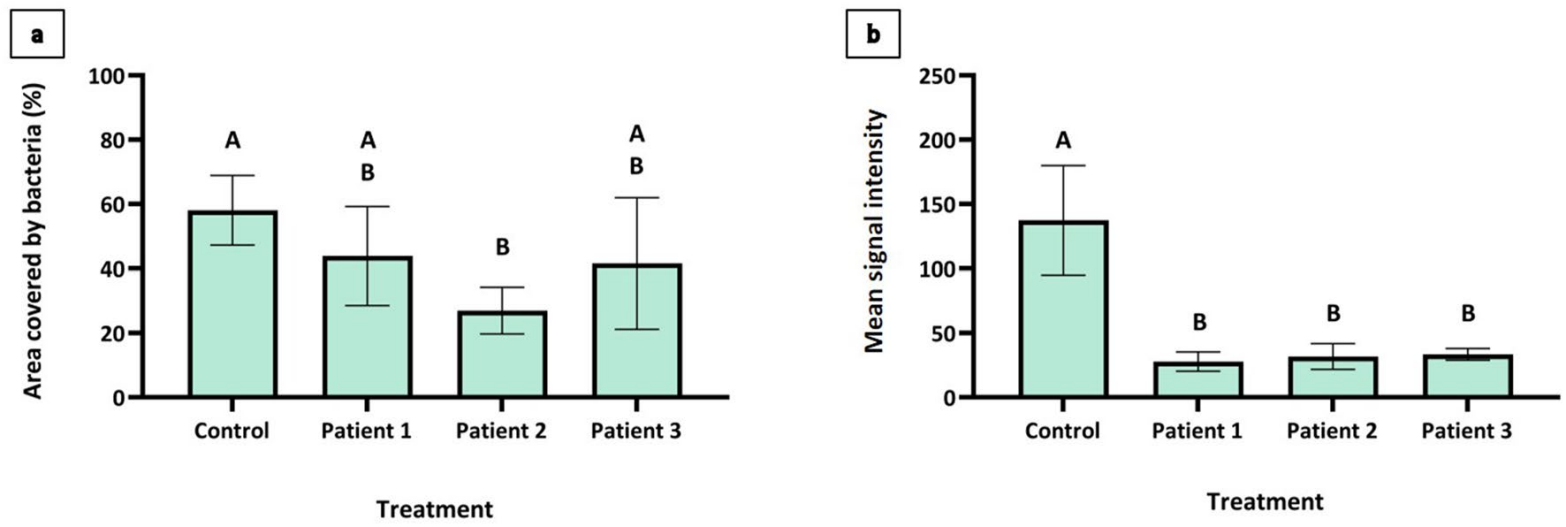
Mean signal intensity (**a**) and area covered by bacteria (%) (**b**) of biofilm growth, in vivo, on splints, postinsertion, evaluated by CLSM. Pieces of ZnCl_2_-coated splints from three patients’ noses (the treatment patients) and non-ZnCl_2_-coated splints from the noses of two patients who were given prophylactic antibiotics (the negative growth control) were stained with PI for CLSM evaluation. The splint pieces were evaluated for the mean signal intensity, quantifying the amount of bacterial growth onto the splint’s surface (**a**) and the area covered by bacterial growth (%) in each piece (**b**). The results that are not significantly different from each other according to Tukey's honestly significant difference (HSD) test are grouped under the same letter (p < 0.05).

**Figure 4:**
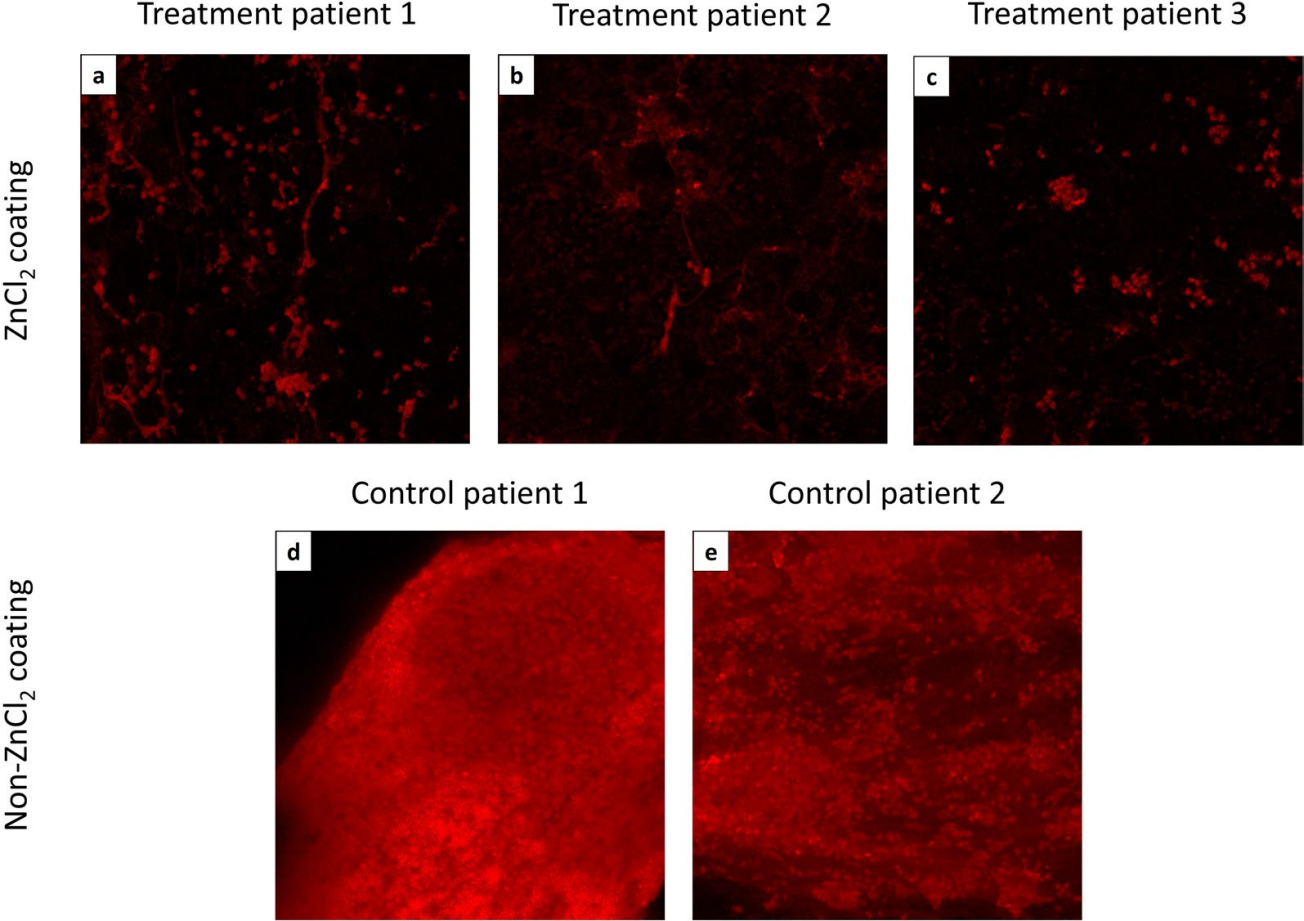
Confocal productions of bacterial biofilm, in vivo, on splints, post insertion, as evaluated by CLSM. Pieces of ZnCl_2_-coated splints from three patients’ noses (the treatment patients) (**a**–**c**) and non-ZnCl_2_-coated splints from the noses of two patients who were given prophylactic antibiotics (the negative growth control) (**d**,**e**) were stained with PI and evaluated by CLSM.

After removal of ZnCl_2_-coated splints from the nasal cavities of three patients, splint pieces were assessed using CLSM for the area covered by bacteria (%), quantifying the ability to attach to the splint’s surface, and for the mean signal intensity, quantifying the amount of biofilm mass. For negative growth control, we used non-ZnCl_2_-coated splints that are in regular use from the nasal cavities of two patients who were given antibiotic prophylaxis for 7 days prior to the removal of the splints and evaluated by CLSM using the same criteria. All treatment patients exhibited a significant decrease (p < 0.05) of over 67% in the mean signal intensity in the ZnCl_2_-coated splints in comparison with control patient 1 (Figs. 3a, and 4). No significant differences were found, however, between the treatment patients and control patient 2. In the area covered by bacteria (%), we found no significant differences between the three treatment groups (Fig. 3b). Significant (p < 0.05) decreases were found when comparing the area covered by bacteria (%) between control patient 1 and treatment patients 2 and 3 and between control patient 2 and treatment patient 3.

### Biofilm mass inhibition of the clinical bacteria on the coated splints in vitro, evaluated by SEM

**Figure 5:**
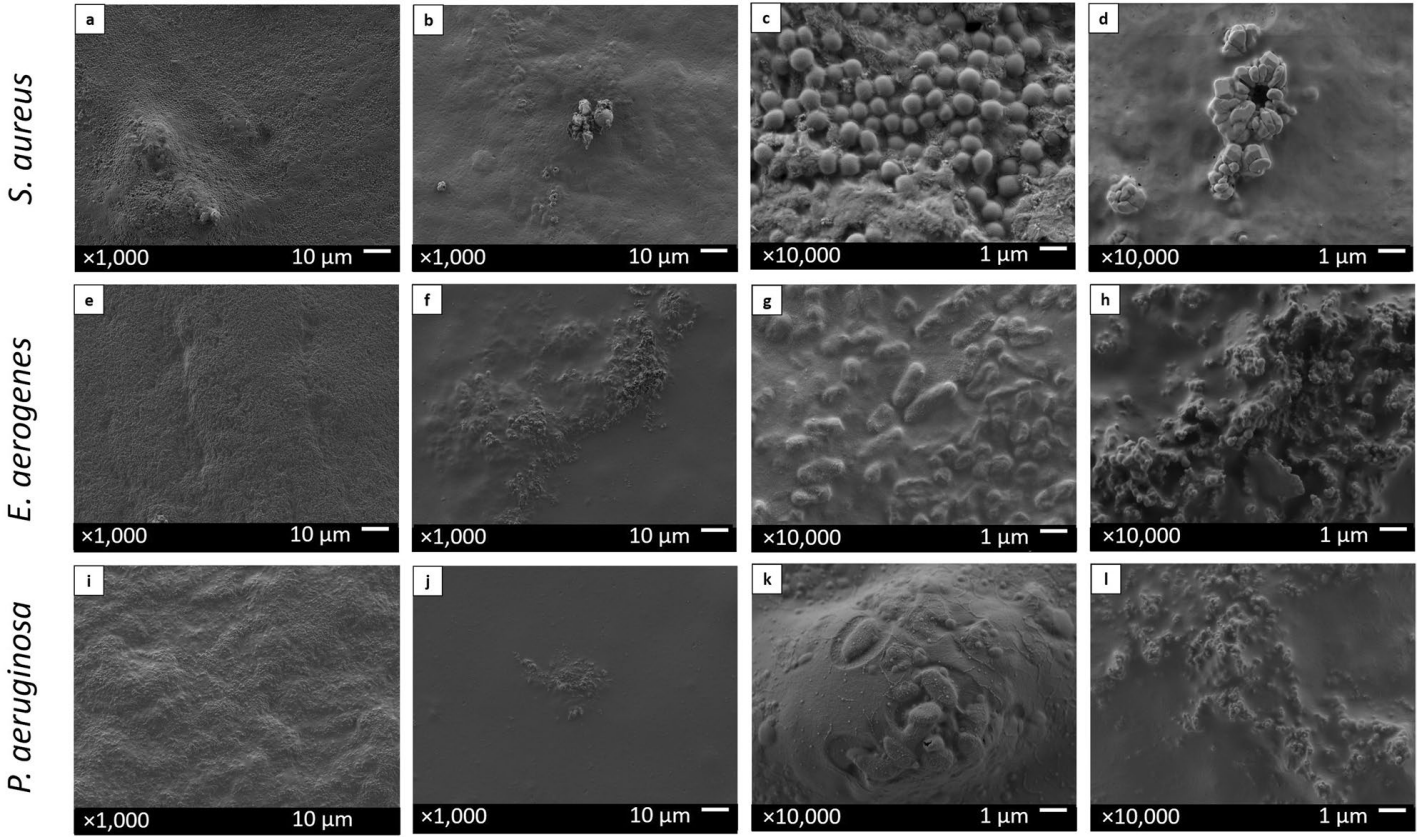
Biofilm growth of clinical bacteria on ZnCl_2_-coated splints and on non-ZnCl_2_-coated splints, captured by SEM. *Staphylococcus aureus* (**a**–**d**), *Enterobacter aerogenes* (**e**–**h**) and *Pseudomonas aeruginosa* (**i**–**l**) were grown for 48 h on ZnCl_2_-coated splints (**b**,**d**,**f**,**h**,**j**,**l**) and on positive growth control non-ZnCl_2_-coated splints (**a**,**c**,**e**,**g**,**i**,**k**). Images were taken with an accelerating voltage of 2 kV at magnifications of 1000 (**a**,**b**,**e**,**f**,**i**,**j**) and 10,000 (**c**,**d**,**g**,**h**,**k**,**l**).

Biofilm formation of three different clinical bacterial strains was evaluated using scanning electron microscopy (SEM). *S. aureus*, *E. aerogenes* and *P. aeruginosa* were inhibited when the bacteria were grown onto the ZnCl_2_-coated splints in comparison with the positive growth control of non-ZnCl_2_-coated splints (Fig. 5). All bacterial strains exhibited low bacterial loads on the ZnCl_2_-coated splints and high bacterial loads on the non-ZnCl_2_-coated splints.

### Biofilm mass inhibition in postinsertion ZnCl_2_-coated splints, in vivo, evaluated by SEM

**Figure 6:**
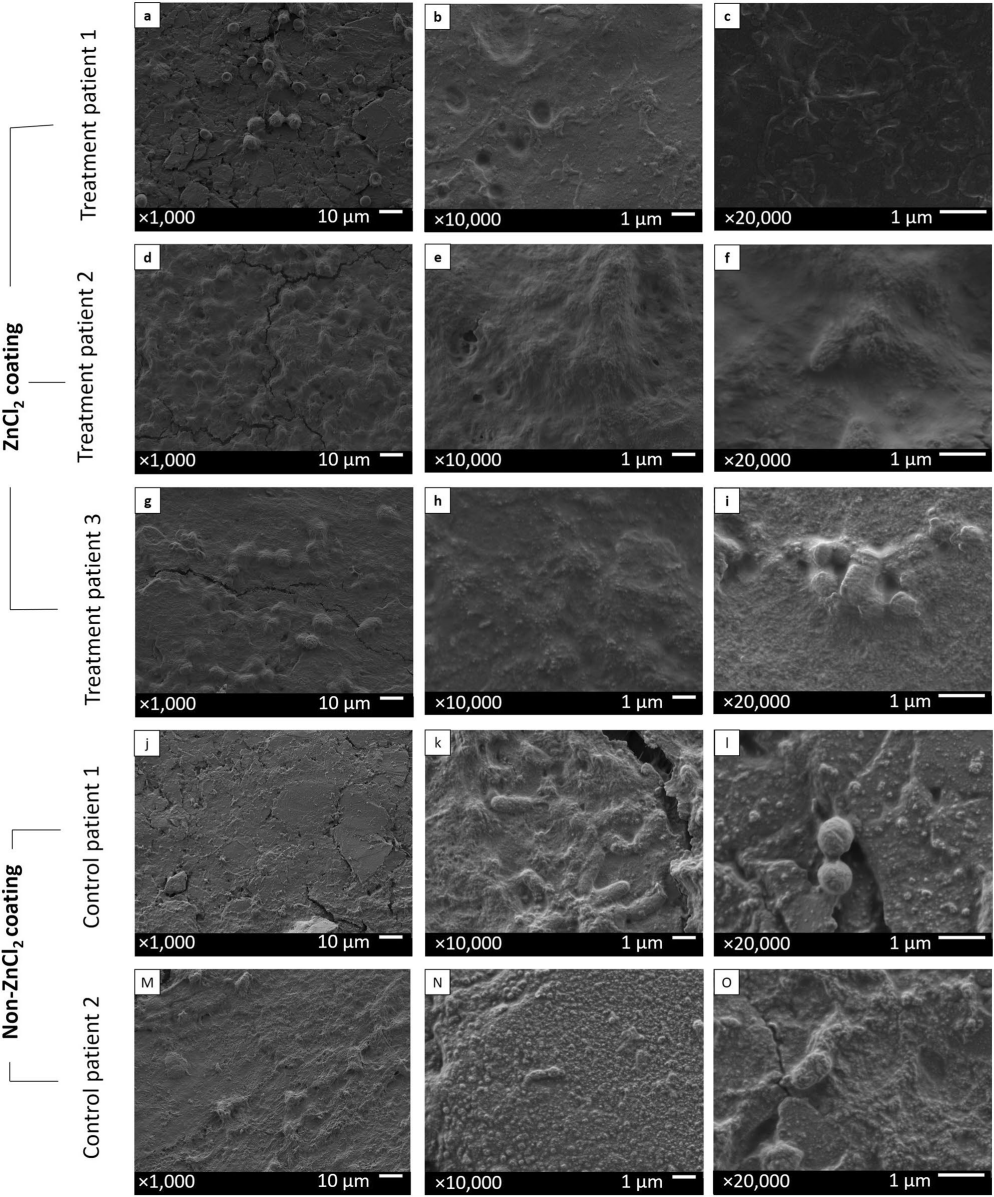
Splints taken from the clinical patients after a 7-day period in the patients’ nasal cavities, captured by SEM. ZnCl_2_-coated splints (**a**–**i**) were inserted into the nasal cavities of three patients for a 7-day period following septoplasty. Regular non-ZnCl_2_-coated splints (**j**–**o**) were inserted for a period of 7 days into the nasal cavities of two patients under prophylactic antibiotic treatment and were used as negative growth controls. Splints were removed from the patients’ nasal cavities and captured by SEM; treatment patient 1 (**a**–**c**), treatment patient 2 (**d**–**f**), treatment patient 3 (**g**–**i**), control patient 1 (**j**–**l**) and control patient 2 (m–o). Images were taken with an accelerating voltage of 2 kV at magnifications of 1000 (left column), 10,000 (middle column), and 20,000 (right column).

All ZnCl_2_-coated splints, extracted after a period of 7 days in the nasal cavities of three treatment patients, exhibited low bacterial load (Fig. 6a–i), along with blood cells that remained attached to the splint’s surface after surgery (Fig. 6a). Non-ZnCl_2_-coated splints from control patient 1 (Fig. 6j–l) exhibited medium bacterial load, with two types of bacterial colonies: bacillus (Fig. 6k) and a coccus (Fig. 6l). Control patient 2 (Fig. 6m–o) exhibited a low bacterial load.

### Toxicity assessment in vivo

Neither pathological clinical signs nor histological differences in the nasal mucosa were observed between the ZnCl_2_-coated splint and non-ZnCl_2_-coated splint treatment groups (data not shown).

## Discussion

The abuse and misuse of antibiotics in recent decades have led to a rapid and worrying rise in the range of antibiotic-resistant bacterial strains, resulting in a strong need to search for alternative antibacterial substances^2,22,34^. Although not universally agreed upon as effective, antibiotic prophylaxis in septoplasty is a common procedure for the prevention of surgical site-related infections^11,20^. In a recent study^11^, bacterial growth was shown to occur after ISS insertion regardless of prophylactic antibiotic therapy, along with an increase in antibiotic-resistant bacteria, actively demonstrating the urgency to replace the use of antibiotic prophylaxis as a preventive treatment in septoplasty. Therefore, we propose to obviate the use of antibiotics by introducing non-antibiotic, antibacterial ZnCl_2_-coated splints as a replacement for the regular ISS. To the best of our knowledge, this is the first study of a natural antibacterial coating for ISS tested in vivo without the administration of antibiotics.

In the present study, we examined the utilization of ZnCl_2_-coated nasal splints as preventative antibacterial treatment against infections post septoplasty surgery, both in vitro and in vivo. In our study, we found (1) a decrease in bacterial growth between the treatment patients and the negative growth control patients when ZnCl_2_-coated splints were placed in patients’ nasal flora without the use of prophylactic antibiotics and (2) ZnCl_2_ was able to inhibit bacterial growth of pathogens considered dangerous antibiotic-resistant bacteria.

An important finding of this study is that ZnCl_2_ was able to inhibit biofilm growth of all three clinically isolated pathogens, *Staphylococcus aureus, Pseudomonas aeruginosa* and *Enterobacter aerogenes*, which are all considered by the WHO to be great threats to humans as antibiotic resistant bacteria and were given the highest “priority status” as to the urgency of finding new antibiotics against them^34^. While the biofilm growth of all of the tested pathogens was inhibited, higher concentrations of ZnCl_2_ were needed for gram-negative bacteria than for gram-positive bacteria. Thus, gram-positive *Staphylococcus aureus* biofilm growth was inhibited at lower ZnCl_2_ concentrations of 0.9 mM to 1.0 mM, while inhibition of biofilm growth in gram-negative *Pseudomonas aeruginosa* and *Enterobacter aerogenes* required higher ZnCl_2_ concentrations of 2.0 mM to 4.0 mM (Table 1). This finding is unsurprising, as gram-negative bacteria are more resistant than gram-positive bacteria, and only a very small portion of the compounds developed against gram-positive bacteria have activity against gram-negative bacteria^35^. Therefore, the ability of ZnCl_2_ to inhibit both gram-positive and gram-negative bacteria is an important finding of this study.

In vitro results exhibited a significant (p < 0.001) decrease of 55–70% in biofilm formation of the clinical bacteria onto the ZnCl_2_-coated splints in comparison with the positive growth control of non-ZnCl_2_-coated splints (data not shown). The inhibition lasted for the entire 168 h of testing, which demonstrates possible effective inhibition of biofilm growth onto the splints throughout their week-long stay in the patients’ nasal cavities. Polymer-coated splints, without ZnCl_2,_ and non-coated commercialized splints showed no significant difference in the biofilm formation onto the two different splints, suggesting that the polymer coating’s surface structure had no significant effect on the bacteria’s ability to attach to the surface. Prior to efficacy testing in vivo, toxicity assessment in rats was performed, where we found no pathological clinical signs or histological differences in the nasal mucosa between the ZnCl_2_-coated splint treatment group and the non-ZnCl_2_-coated splint control group (data not shown).

When toxicity was ruled out, we continued to examine the efficacy in vivo by inserting ZnCl_2_-coated splints into the nasal cavities of three patients. To obtain a quantitative assessment, rather than a qualitative one, we performed a microscopic examination of the splints using CLSM. Although not significantly different between all patients, the ability of the bacteria to attach to the ZnCl_2_-coated splint surfaces was lower than in the regular non-ZnCl_2_-coated splints obtained from patients who received prophylactic antibiotic treatment, representing negative growth control (Fig. 5). We did find, however, a significant (p < 0.05) decrease of 66–72% in the mean signal intensity, quantifying the amount of biofilm mass, between control patient 1 and the three treatment patients and a non-significant decrease of 57–64% in the amount of biofilm mass between the three treatment patients and control patient 2. This is to be expected, as the probability of a biofilm-related infection to occur on a polymeric implant surface is between 65 and 80%^36^. However, even though the bacteria were able to attach to the surface, the ZnCl_2_ coating inhibited biofilm growth onto the splint surfaces during the week-long stay in the patients’ nasal cavities, resulting in effective treatment against ISS insertion-related infections without the need for prophylactic antibiotics, with the treatment being at least as effective as with antibiotics.

In conclusion, from our preliminary study, it appears that ZnCl_2_ is a natural, effective and inexpensive solution that is able to inhibit biofilm growth of pathogens that are considered hazardous as antibiotic-resistant bacteria while obviating the use of prophylactic antibiotic therapy following insertion of ISS as part of septoplasty surgery. It should be taken into consideration that although statistically significant, to implement this solution in clinical practice, a greater number of subjects should be examined, and technical improvements should be made to the coating (more even spread, improved ease of use, longer lasting coating).

## Methods

### Bacterial strains and growth conditions

The bacteria used in this study were obtained from the microbiology department of Hasharon Hospital (Petah Tikvah 4937211, Israel), isolated, and kept at − 80 °C*.* All of the bacteria were isolated from the nasal flora of patients treated and not treated with antibiotics after removal of the ISS (Table 2). All strains were propagated in lysogeny broth (LB) (Difco) or in solid LB medium comprising 1.5% Bacto-Agar (Difco). The LB broth contained, per litre, 10 g of tryptone, 5 g of yeast extract and 5 g of sodium chloride (NaCl). For routine growth, we grew each strain on solid LB for 24 h at 37 °C. Later, a starter culture was prepared by inoculating one colony of each strain into 5 mL of liquid LB. Starter cultures were then incubated overnight at 37 °C while shaken at 100 rpm. The starter was then either further incubated or diluted in the broth until an OD_600 nm_ of 0.50 was obtained, measured using spectrophotometry (WPA CO8000, Biochrom, Cambridge, United Kingdom) and then diluted 1:100 into the relevant experiment medium. For planktonic growth, we used liquid LB, and for biofilm growth, we prepared LBGM by supplementing LB with 1% (v/v) glycerol and 0.1 mM manganese sulfate (MnSO_4_)^33^.

**Table 2:** Bacterial isolates used in the in vitro study from participating patients.

Age	Gender	Date of sample	Isolate ID	Identified bacteria	Antibiotics treated
43	Male	21.1.2021	43021480	*Staphylococcus aureus*	Yes
			43021481	*Staphylococcus aureus*	
25	Female	27.5.2021	43013795	*Enterobacter aerogenes*	No
			43013796	*Enterobacter aerogenes*	
57	Male	27.5.2021	43013791	*Pseudomonas aeruginosa*	No
			43013792	*Pseudomonas aeruginosa*	

Identification of the strain was accomplished by using the 16S ribosomal RNA sequencing method^37^ using the GeneJET Gel Extraction and DNA Cleanup Micro Kit (Thermo Scientific) and the 27F and 1492R primers^38^. Standard DNA sequencing of the samples was performed by the Genomic Technologies Facility in The Alexander Silberman Institute of Life Sciences, Hebrew University of Jerusalem. For *Staphylococcus aureus* and *Enterobacter aerogenes*, an OD_600 nm_ of 0.50 ≈ 1 × 10^8^ CFU/ml, and for *Pseudomonas aeruginosa*, an OD_600 nm_ of 0.50 ≈ 1.5 × 10^8^ CFU/ml.

Zinc chloride (ZnCl_2_) (Sigma-Aldrich, St. Louis, MI, USA) was used as the antibacterial substance in our study in a 1 M concentrated solution that was added to the growth medium in different dilutions until reaching the desired variety of final concentrations.

### ISS coating method

The coated nasal splints used in this study were obtained from Professor Michael Friedman’s laboratory (School of Pharmacy, the Faculty of Medicine, the Hebrew University of Jerusalem). Polyethylene glycol 400, a desired solvent volume of aqueous ethanol solution, was filled into a chemical beaker of a suitable size equipped with a magnetic stirring rod. The contents were mixed at approximately 35 °C at a velocity that produced a stable vortex. Klucel™ hydroxypropyl cellulose HF was then gradually added to ensure proper wetting and mixed at 25 °C to 35 °C until complete dissolution of the polymers, assessed visually against a clear glass slide. ZnCl_2_ was then added to the polymer solution and mixed until dissolution or until stable dispersion was obtained. The flow properties were evaluated by discharging 1 mL of the formulation from a syringe without a needle. The coating finally contained 0.3 g of polyethylene glycol 400, 0.7 g of Klucel™ hydroxypropyl cellulose HF and 0.5 g of ZnCl_2_ in 30 cc ethanol/H_2_O 9:1 and 1.5 mL H_2_O. Silicone splints (Grimaldi nasal splint, N5, Exmoor Plastics Ltd, United Kingdom) were coated on each side by pipetting the formulation, evenly spreading and then drying. Excess coating was removed, or additional coating was added to ensure uniform weight of the coating. Prior to performing experiments with the splint pieces, they were disinfected using ultraviolet (UV) radiation for 1.5 h on each side.

### Determination of ZnCl_2_ antibacterial efficacy against planktonic cells of the clinical bacterial isolates

MIC is defined as the lowest concentration of a substance that inhibits the visible growth of a planktonic culture. MBC is defined as the lowest concentration of a substance that reduces the initial inoculum of a planktonic culture by 99.9%^39^. To determine the MIC value, we used the broth dilution method on the examined bacterial strains, as described in Balouiri et al.^40^ with slight modifications. Briefly, a starter culture was generated for each isolate and then diluted to 1:100 into a 48-well plate, and ZnCl_2_ was added into each well in gradual concentrations, starting at 0.5 mM and up to 10 mM. Plates were then incubated overnight at 37 °C and examined for growth the following morning. MIC was determined according to the wells in which there was no visible growth, and the concentration of ZnCl_2_ was the lowest. To determine the MBC value, 100 µL of each of the wells with higher concentrations than the determined MIC value were plated on solid LB for 24 h at 37 °C, as previously described^40^. Then, the plates were counted for living cells (CFU/mL), and MBC values were determined as the lowest concentration at which 99.9% of the initial inoculum was killed. For concentrations within the tested range in which these criteria were not met, MIC and MBC were determined as not found (NF). An assay was performed in three independent experiments, each performed in triplicate for each ZnCl_2_ concentration.

### Quantification of ZnCl_2_ effect on biofilm formation

BPC is defined as the lowest concentration of an antibacterial substance that causes a 1 log reduction in biofilm growth at OD_650 nm_, with simultaneous exposure of the bacterial inoculation and the antibacterial substance^39^. To determine the BPC value, we performed a susceptibility assay as previously described^39^, with some changes. A starter was diluted to 1:100 in LBGM medium and placed in a 96-well plate (round-bottom, Nunc™) along with gradual concentrations of ZnCl_2_ from 0.1 to 0.9 mM in 0.1 mM steps and 1.0–4.0 mM in 1.0 mM steps, with four wells for each ZnCl_2_ concentration. After 24 h of incubation at 37 °C in static conditions, 100 µL of each well was transferred to a new 96-well plate (flat-bottom, Nunc™), and the OD_595 nm_ was measured using a plate reader (ELx808™, Bio-Tek instruments, Vermont, USA). BPC was determined according to the wells with the lowest concentration that yielded at least a 1 log difference in growth. An assay was performed in three independent experiments, each performed in quadruplicate for each ZnCl_2_ concentration.

### Assessment of coated splint efficacy against biofilm formation of clinical bacteria using crystal violet (CV) staining

One-cm^2^ pieces of the ZnCl_2_-coated splints were placed in a 24-well plate (Nunc™), along with pieces of coated splints without ZnCl_2_ and uncoated splints as a positive growth control. We diluted a generated starter to 1:100 in LBGM medium and then pipetted 150 µL on the upper side of each of the coated splint pieces. Later, the plate was incubated in static conditions at 37 °C, and the splint pieces were checked for biofilm growth after 10 h, 24 h, 48 h, 72 h and 168 h. Biofilm quantification was accomplished by the microtiter dish biofilm formation assay, as used previously^41^, with modifications; the splint pieces were washed twice in distilled water to remove planktonic cells and then incubated in 0.1% (w/v) CV solution for 15 min. Later, the stain residue was washed using distilled water, and stained pieces were left to dry overnight at room temperature (RT). Next, we added 30% acetic acid to solubilize the CV for 15 min (RT). Extracts were then placed in a new flat-bottom 96-well plate (Nunc™) and measured using a plate reader (ELx808™, Bio-Tek instruments, Vermont, USA).

### Assessment of coated splint efficacy against biofilm formation using SEM

#### Sample preparation

For microscopic visualization of biofilm growth on the splint surfaces, we placed 1 cm^2^ pieces of noncoated splints, coated splints without ZnCl_2_ and ZnCl_2_-coated splints in a 24-well plate (Numc™). We diluted a generated starter to 1:100 in LBGM medium and then pipetted 150 µL on the upper side of each of the splint pieces. Then, the plate was incubated in static conditions at 37 °C. After 24 h of incubation, 150 µL of fresh diluted starter was again pipetted onto the upper side of each splint’s surface, and the plate was incubated for another 24 h at 37 °C for a total of 48 h. Later, the pieces were washed and fixated according to the bacterial strain, as described previously^42^, with slight modifications as detailed below. A 4% glutaraldehyde solution was used as a fixative by diluting an 8% glutaraldehyde stock (SPI Chem, Pennsylvania, USA) 1:1 with double distilled water (DDW). A 4% glutaraldehyde solution was preferred to a common 2.5% glutaraldehyde and 2.5% formaldehyde solution, since the experiment focused on surface morphology rather than internal structure. Since ethanol caused the total removal of the aqueous-based biofilm sheet, post-fixation dehydration was performed using air-drying instead.

For *Staphylococcus aureus,* the pieces were fixated immediately after the incubation period, without preliminary washes, by adding 300 µL of 4% glutaraldehyde to each well. Following one hour of incubation, the pieces were washed once in 400 µL of phosphate-buffered saline (PBS) and once in 400 µL of DDW, 10 min each in static conditions (RT). Finally, the pieces were left to air-dry overnight (RT). Samples were kept at 4 °C until microscopy evaluation.

For *Enterobacter aerogenes* and *Pseudomonas aeruginosa*, after 48 h of incubation, unattached cells were removed by washing the pieces twice in 400 µL of PBS and once in 400 µL of DDW, 10 min each in static conditions (RT). Then, the pieces were fixated by adding 300 µL of 4% glutaraldehyde to each well for a one-hour incubation in static conditions (RT). Following incubation, the pieces were washed twice in 400 µL of DDW, 10 min each, under static conditions (RT). Finally, the pieces were left to air-dry overnight (RT). Samples were kept at 4 °C until microscopy evaluation.

#### Microscopy

Prior to the microscopy evaluation, all of the splint pieces, regardless of the bacterial strain, were cut into four even pieces, coated with a 1 nm gold layer (Au/Pd) (QUORUM Q150T ES) and then visualized by SEM (JEOL, JSM-7800F, Tokyo, Japan Schottky Field Emission Scanning Electron Microscope). The images were taken with an accelerating voltage of 2 kV at magnifications of 1,000, 10,000 and 20,000.

#### Grading of treatment efficacy

To grade the treatment efficacy, images were characterized as high microbial load (Fig. 5a), medium microbial load (Fig. 6k) and low microbial load (Fig. 5b).

### Assessment of coated splint efficacy against biofilm formation using CLSM

#### Sample preparation

For CLSM visualization of biofilm growth on the splint surfaces, the same growth and fixation protocol was conducted as in the preparation for assessment of coated splint efficacy against biofilm formation using SEM. Prior to the CLSM evaluation, the fixated splint pieces were stained with propidium iodide (PI) (Rhenium) by diluting 130 µL of PI in 870 µL DDW to create 1 mL of stock solution, stored in tin foil at 4 °C, and further diluting the PI stock solution 1:10 in DDW. Then, 300 µL of the diluted PI solution was added to each well. This was followed by incubation for 30 min while covered in tin foil (RT). Following incubation, the splint pieces were washed with 400 µL of PBS and were left with the PBS solution at 4 °C, covered in tin foil until microscopy evaluation, as described previously^42^. PI was preferred to CV based on past experience with catheters in our lab, and given that all bacteria were non-living following the fixation step.

#### Microscopy

The biofilm growth on the splint surfaces was evaluated by CLSM (LEICA SP8), and the fluorescence intensity was measured using dry 20×/0.7 NA objective lens with zoom factor of 1.28, excitation of 561 nm and emission of 600–650 nm, with PMT gain of 850 [V]. Pixel format 1024 × 1024, voxel size 0.446 × 0.446 × 2 µm (X, Y, Z, respectively) (Supplementary Information).

#### Image analysis

Image processing and analysis were accomplished in the following steps: (1) the saved '.lif' file was saved as individual '.tif' files, with a Fiji macro “ImageJ_Export-LIF-as-Individual-Images-master_pmascalchi”^43^, and (2) the files were processed and analyzed with a second macro “area_fraction_analysis.ijm”^44^, in eight different areas per tested sample.

### Assessment of coated splint toxicity in vivo

We investigated the safety of using ZnCl_2_-coated splints in the prevention of nasal mucosal infection in rats. The safety study was approved by the Beilinson Ethics Committee and conducted in the Frankel experimental research laboratory at the Felsenstein Medical Research Center (Petah Tikva, 4,941,492, Israel). Eighteen male and female Sprague–Dawley (SD) rats were used in this study and were maintained according to standard guidelines. A silicone internal nasal splint (INS) and a ZnCl_2_ silicone-coated splint (Z-INS) were cut into 1 mm × 0.85 mm × 7 mm pieces. The rats were randomized into a control group, implanted with INS (n = 6), and a treatment group, implanted with Z-INS (n = 12). The splints were placed in the right nasal passages of the rats for 7 days. Then, tissues were decalcified, trimmed, embedded in paraffin, sectioned and sent for histological processing, conducted by the research unit of PATHO-LAB Diagnostics Ltd. (Ness Ziona, Israel). Histological evaluation was obtained using a BX43 Olympus light microscope and DP21 Olympus digital camera with Olympus cellSens Entry 1.13 software. Samples were evaluated according to known parameters, as described in Şevik Eliçora et al*.*^45^.

### Assessment of coated splint efficacy in vivo

Following nasal surgery, surgeons from Hasharon Hospital (Kakal Street 7, Petah Tikva, Israel) inserted ZnCl_2_-coated splints into the nasal cavities of three patients in lieu of the conventional uncoated splints. The splints were left in the patients’ noses for 7 days postsurgery and then removed, washed in PBS and fixed with 4% glutaraldehyde. The splints were left to set for one hour and then washed twice for 10 min each in PBS under static conditions (RT). The splints were left to air-dry overnight (RT) and finally kept at 4 °C until microscopy evaluation.

The patients who participated in the study abstained from using regular medications, antibiotic treatment, nasal sprays and nasal corrosion agents during the month prior to the surgery.

### Ethics

Ethical approval for the animal study and for all experimental protocols was granted by the Rabin Medical Center ethics committee (Helsinki) for research on the effects of polymer-based, slow-release zinc-coated devices for the prevention of infection in animal models. Approval number: 021219 for a 4-year period, starting 01/12/19. All methods were carried out in accordance with relevant guidelines and regulations and are reported in accordance with ARRIVE guidelines.

Ethical approval for the human study was granted by the Rabin Medical Center ethics committee (Helsinki) for a phase 1 trial in humans for research on using ZnCl_2_-coated splints to prevent bacterial growth on nasal splints. Approval number: 0374-19-RMC for a 1-year period, starting 23/9/2021. All research was performed in accordance with relevant guidelines and regulations, and informed consent was obtained from all subjects and/or their legal guardian(s).

### Statistical analysis

The obtained numerical data were analyzed statistically by means of ANOVA following a post hoc t-test using JMP software at significance p-values < 0.05. The results are based on three biological experiments performed in triplicate.

## Supplementary Information

Supplementary Information.

## Data Availability

The data that support the findings of this study are available from the corresponding author upon reasonable request. The datasets generated and/or analyzed during the current study are available in the NIH repository, accession numbers: OQ195154, OQ195155, OQ195156, OQ195157, OQ195158 and OQ195159.
