# Supplementary material for: Zinc chloride is effective as an antibiotic in biofilm prevention following septoplasty
Source: Sci Rep. 2023 May 23;13:8344. doi: 10.1038/s41598-023-35069-9 (PMC10206080; doi:10.1038/s41598-023-35069-9)
Supplement: Supplementary file 1 — Supplementary Information. [file 41598_2023_35069_MOESM1_ESM.pdf]

Zinc chloride is effective as an antibiotic in biofilm prevention following septoplasty

Noa Noach., Eran Lavy DVM., Ram Reifen MD., Michael Friedman., David Kirmayer., Einat Zelinger., Amit Ritter MD., Dan Yaniv MD. & Ella Reifen MD.

Image: **control splint 1**

Size: **10.49 MB**

File Location: **E:\Confocal\Ram Reifen\Noa\22.8.14 Noa.lif**

Start Time: **2022-08-14 10:10:54.360 AM**

End Time: **2022-08-14 10:11:01.140 AM**

Total Exposures: **10 (1 channels, 10 frames)**

Data from: **LAS X 3.5.5.19976**

Dimensions

Dimension	Logical Size	Physical Length	Start Position	End Position	Pixel Size / Voxel Size
X	1024	455.88 µm	0 µm	455.88 µm	0.446 µm
Y	1024	455.88 µm	0 µm	455.88 µm	0.446 µm
Z	10	7.08 µm	-50.55 µm	-43.47 µm	0.787 µm

Channels

LUT	Resolution	Min	Max	STED: DetectorMode / Huygens saturation factor / Wavelength
Red	8	0	255	--- / --- / ---

Time Stamps:

Frame (Show All)	Relative Time (s)	Absolute Time (h:m:s.ms)	Date
1	0.000	10:10:54 AM.360	2022-08-14
10	6.780	10:11:01 AM.140	2022-08-14

Confocal Settings

Name	Value
Rotator Angle	0 °
Scan Mode	xyz
Scan Direction X	Bidirectional
Scan Speed	700 Hz
Version Number	15
StagePosX	100,223.15 µm
StagePosY	41,763.24 µm
ZPosition	-43.47 µm
IsSuperZ	0
Magnification	20

ObjectiveName	20x/0.70 DRY
Immersion	DRY
Numerical Aperture	0.7
RefractionIndex	1
Zoom	1.28
Pinhole	60.6 µm
PinholeAiry	999.76 mAU
EmissionWavelength for PinholeAiry Calculation	580 nm
FrameAverage	1
LineAverage	1
FrameAccumulation	1
Line_Accumulation	1
IsUserSettingNameSet	0
IsRoiScanEnable	0

Filter Wheels / Other Motorized Devices

**Device Name**	**Filter Name/Position**
Excitation Beam Splitter	DD 488/552
Galvo Slider	Galvo X Normal
Notch FW 2	NF 552
Polarization FW	Empty
Galvo Resonant Pan	Galvo X Pan Center
Simple Beam Expander	No FRAP Booster
Target Slider	Target Park
X2 Lens Changer	CS2 UV Optics 1

Lasers

**LaserName**	**OutputPower**
**Diode 405**	**On**
**Diode 638**	**Off**
**OPSL 488**	**Off**
**OPSL 514**	**Off**
**OPSL 552**	**On**

Laser Lines

**Laser Line**	**Intensity**
(405 nm)	Shutter: off, Intensity: 0.0000%
(488 nm)	Shutter: on, Intensity: 0.0000%
(514 nm)	Shutter: on, Intensity: 0.0000%
(552 nm)	Shutter: on, Intensity: 10.0013%
(638 nm)	Shutter: on, Intensity: 0.0000%

Detectors
